# Book Review

**DOI:** 10.1556/JBA.3.2014.4.8

**Published:** 2014-12-18

**Authors:** Máté Kapitány-Fövény

**Affiliations:** Doctoral School of Psychology, Institute of Psychology, Eötvös Loránd University, Budapest, Hungary E-mail: kapitany.mateppk.elte.hu

**Figure 1:**
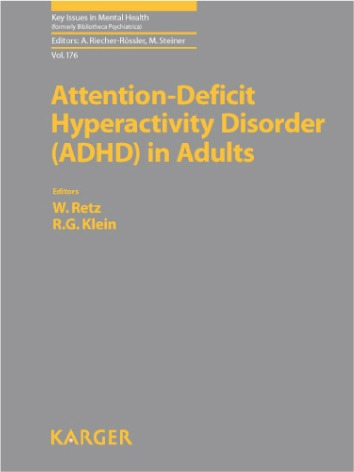


Attention-deficit hyperactivity disorder (ADHD) is considered to be one of the main risk factors for the onset and persistence of substance use disorders (SUD). Based on the work of – for instance – Joseph Biederman, Timothy Edwin Wilens or Stephen Faraone it has been well established that adults with ADHD abuse psychoactive substances more often, for a longer period of time and at an earlier age, and they show a faster transition from alcohol abuse to illicit substance abuse compared to those without ADHD. Therefore, early detection and treatment of attentional problems, hyperactivity and impulsivity (as distinct components of this disorder) is important in both the prediction and prevention of SUD onset. It is also crucial to broaden social cognition about ADHD as this disorder may have a severe impact not only on substance abuse but also on self-esteem, academic achievement, potential criminal activities or interpersonal relationships as well.

The editors, Wolfgang Retz and Rachel G. Klein aimed to provide an overview of the most relevant aspects of ADHD – epidemiology, neurobiology, psychopathology, longitudinal course, comorbidity and social impairment – across lifespan, following the best traditions of *Key Issues in Mental Health*. The authors are ADHD-experts from the USA and Germany representing various scientific fields, such as psychiatry, psychology, neurology and genetics.

Paul H. Wender and David A. Tomb present ADHD’s etiology, treatment options and symptomatology in Chapter 1, including childhood and adulthood characteristics of the disorder as well as highlighting core symptoms that ease differential diagnosis. Authors also provide a brief overview of the history of the ADHD Concept. The part where authors introduce case histories as well as the Appendix of several ADHD rating scales will surely be well-appreciated given their potential benefit in both clinical and scientific work.

Christine Margarete Freitag and Wolfgang Retz delineate genetic background of ADHD in Chapters 2 and 3. Readers learn about the heritability of ADHD subtypes, the comorbidity of this disorder – Oppositional Defiant Disorder, Conduct Disorder, Major Depressive Disorder and Generalized Anxiety Disorder as most frequently occurring comorbid disorders –, sex differences based on the results of longitudinal twin, family and adoption studies. Molecular genetics of ADHD is presented through the findings of association studies (genomewide association studies, for instance). The role of the dopamine transporter and receptor genes – which play a significant role in the development of SUD too – as well as the dopamine-catalyzing enzymes, noradrenergic genes and serotonergic genes is highlighted by the authors.

In Chapter 4, neurophysiology of ADHD is described by Christina G. Baehne and Andreas J. Fallgatter. Current methods of electrophysiological measures – such as EEG and quantitative EEG (qEEG) – are illustrated. Elevated theta power and reduced alpha and/or beta power in ADHD children or lower P3 amplitudes as part of event-related potentials are well-studied neurophysiological characteristics of the disorder.

Marc Schenider, Michael Rösler and Wolfgang Retz introduce the findings of brain imaging studies in Chapter 5. Brain region-specific results – the role of the frontal, temporal, parietal and occipital lobes and more specific areas, including basal ganglia or corpus callosum – and ADHD-related brain functioning – the subcortical location of arousal and alerting networks as an example – are also described here.

Rolf-Dieter Stieglitz in Chapter 6 introduces diagnostic criteria and prevalence rates of ADHD showing some overlap in content with Chapter 1. In addition to this, however, the multimodal assessment and the diagnostic process which are presented here with several practical recommendations make this chapter more than helpful for clinicians and psychologists. For instance, Figure 1, that presents the diagnostic process with diagnostic aids such as when to use screening instruments, structured interviews or rating scales during this process, is a truly useful guideline for clinicians.

A full chapter is dedicated to the comorbidity of ADHD with other disorders and was written by Rachel G. Klein and Salvatore Mannuzza (Chapter 7). Here the volume is slightly repetitive again, even if SUD is firstly mentioned by Klein and Mannuzza as a typical comorbid disorder of ADHD. Methodological and diagnostic difficulties are also addressed in this chapter.

The correlation between ADHD and criminality is exposited in an intriguing chapter by Michael Rösler. Functional impairments that lead to criminal activities and dysfunctions in the everyday life of ADHD patients – e.g. earlier onset of sexual activities, lack of parenting skills or high risk of accidents – are described. Several figures and tables make this chapter easier to follow, while individual and social issues related to ADHD are superbly outlined.

Alexandra Philipsen, Harald Richter, Swantje Matthies and Bernd Hesslinger present psychotherapeutic possibilities in Chapter 9. In the psychological treatment of ADHD cognitive behavioral therapy (CBT) is well-known, authors, however, introduce many more, less familiar therapeutic approaches, such as disorder-oriented group and individual programs. The goals of these approaches are demonstrated by describing the Freiburg Treatment Program. This program aims to enhance patients’ problem-solving skills and affect regulation using mindfulness techniques, behavior analysis, impulse control tasks, involving partners and family members as well.

In the final chapter, Götz-Erik Trott lists available psychopharmacological treatment options, starting with a brief historical overview of the therapeutical use of benzedrine and methylphenidate. Trott emphasizes that stimulant medications are still the first-line treatment options for the pharmacotherapy of ADHD, although the use of anti-depressants and alpha-adrenergic agents is also a significant part of pharmacological treatment. For adult ADHD patients the use of atomoxetine as a non-stimulant medication is found to be one of the best pharmacotherapeutical options. The volume is a complex and up-to-date summary of the many aspects of ADHD. Its focuse on adulthood (as opposed to childhood) is favorable given that ADHD was handled and considered as a childhood disorder for many years, therefore individual and social impairments of ADHD in adulthood had been largely neglected. The main strength of this book lies in its capability to provide useful information not only for the experts of this field but also for lay individuals who are either directly or indirectly impacted by ADHD in their lives, or seek answers to their questions related to this disorder. *Attention-deficit hyperactivity disorder (ADHD) in adults* is a plainly recommended reading which can also be used as a handy guideline for psychotherapy and pharmacotherapy as well.

